# Changes in health-related rehabilitation trajectories following a major Norwegian welfare reform

**DOI:** 10.1186/s12889-023-16272-9

**Published:** 2023-07-28

**Authors:** Sina Wittlund, Thomas Lorentzen

**Affiliations:** 1 Nordland Hospital Trust, Regional Competence Centre for Work and Mental Health, PO Box 1480, 8092 Bodø, Norway; 2 Department of Community Medicine, UiT, The Arctic University of Norway, PO Box 6050 Langnes, N-9037 Tromsø, Norway; 3 Department of Sociology, University of Bergen, PO Box 7802, 5020 Bergen, Norway

**Keywords:** Mental disorders, Mental health, Health-related rehabilitation, Work Assessment allowance, Social policy, Welfare reform, Educational, work and welfare trajectories, Medicalisation, Disability benefits, Young adults

## Abstract

**Background:**

In this study we investigated the health-related rehabilitation trajectories of young Norwegian adults between 2004–2019. The study period is interesting because it overlaps with an extensive welfare system reform that occurred in Norway between 2006–2011. In parallel with the reform there was a substantial increase in health-related welfare dependency among young people due to mental health conditions.

To better understand this group, we addressed three questions: 1) what were the most typical health-related rehabilitation trajectories for young Norwegians aged 23–27 between 2004–2019, 2) did the trajectories and composition of health-related benefit recipients change overtime and 3) in parallel with the welfare reform, do we see improved labour market outcomes in our study population?

**Methods:**

Using high-quality Norwegian registry data, we established four cohorts of Norwegian health-related rehabilitation benefit recipients aged 23–27 in either 2004 (cohort 1), 2008 (cohort 2), 2011 (cohort 3) or 2014 (cohort 4). The follow-up period for each cohort was six years. We used sequence and cluster analyses to identify typical health-related rehabilitation trajectories. In addition, descriptive statistics and multinomial logistic regression were used to scrutinise the relationship between trajectory types, sociodemographic characteristics and cohort membership.

**Results:**

The majority follow trajectories consisting of welfare dependency, unemployment and unstable, low-income work. Both the trajectories and composition of the study population changed across cohorts. Over the observation period there was a 1) three-fold increase in the proportion following a trajectory ending in permanent disability benefits, 2) nine-fold increase in the proportion following trajectories characterised by long periods of health-related rehabilitation, 3) five-fold decrease in the share following unemployment occupational handicap trajectories 4) 6.9% increase in the proportion of early school leavers and 5) 8.9% decrease in the share with disabled parents.

**Conclusion:**

Our study population is a vulnerable group with suboptimal mental health, functioning and employment outcomes. In conjunction with the welfare reform, we witnessed a significant drop in use of work-related benefits, accompanied by a substantial increase in uptake of health-related rehabilitation- and disability benefits. Thus, it appears that rather than improving employment outcomes, welfare policy changes have created a new problem by steering a greater proportion into disability benefits.

**Supplementary Information:**

The online version contains supplementary material available at 10.1186/s12889-023-16272-9.

## Introduction

### Background

Preventing early labour market exclusion is a global priority [1]. Increasing unemployment and reliance on health-related welfare benefits among young adults from Western countries has led to social exclusion, negatively affecting their health, well-being, and overall quality of life [2]. When young individuals are denied the opportunity to develop their life skills and pursue their aspirations, society loses out on their valuable contributions, resulting in a loss of value creation. A premature departure from the labour market by potentially productive individuals also imposes a considerable economic burden on the public, leading to prolonged social security payments for many years [3].

A concerning trend in several European countries, including Norway, is the growing dependence of young adults on health-related welfare benefits [3, 4]. Various socioeconomic factors, such as lower education or income, limited connection to the workforce, immigrant status, weak social relationships, and family background factors like parental limited education or unemployment, contribute to this risk [5, 6]. Qualitative research in Norway highlights the significance of challenging childhoods, adjustment difficulties, and adverse social experiences like abuse and bullying for young individuals receiving disability benefits [7].

Mental and behavioural disorders significantly contribute to the disability burden experienced by young people in high-income countries [8]. The OECD recognizes mental ill-health as a problem for social and labour market policies, as it results in high costs for individuals, employers, and society due to reduced employment, increased unemployment, and productivity losses [3].

Although the overall unemployment rate remains low in Norway, there is a significant concern about the proportion of young adults relying on social benefits due to health issues [9]. In Norway, approximately 5.2 per cent of young people aged 18–29 receive either temporary health-related rehabilitation benefits or permanent health-related disability pensions [10], considerably higher than in other OECD countries. Young adults aged 25–29 constitute more than half of this group. Of particular concern is the nearly doubled dependency on health-related welfare benefits among young people in the past two decades, especially for those granted benefits due to mental health conditions [10].

### The Norwegian Labour and Welfare administration

Over the past 20 years, changes have taken place in the Norwegian welfare system. Before 2006, three separate agencies provided labour and welfare services in Norway: Employment Services, Social Insurance Administration, and Municipal Social Services. However, between 2006 and 2011, the Norwegian government implemented a major reform by merging these agencies into a new organisation called the Norwegian Labour and Welfare Service (NAV) [11]. This reform aimed to address the challenges caused by the fragmented structure of the previous system [12]. The main goal was to break down administrative barriers and customise services for individual users to help them find employment. The motivation for these changes was concerns about high rates of labour market exclusion and the frequent passing of individuals with multiple problems between different agencies. The overarching objectives of NAV were to increase employment rates and reduce dependence on benefits.

NAV represents what the literature calls a "one-stop shop" [13]. Many OECD countries, including the Netherlands, Austria, and Finland, have also adopted similar approaches of establishing one-stop shops or single gateways for welfare, employment, and social assistance since the late 1990s [14–16]. These reforms aimed to provide comprehensive and user-centred services, simplifying the process for individuals seeking assistance. Despite national differences, the common objective across all countries was to increase workforce participation, particularly for individuals facing significant barriers to (re) entering the labour market [13].

### The work assessment allowance

An important innovation of the NAV reform occurred in 2010 when three separate benefits (temporary disability, vocational rehabilitation and medical rehabilitation) were amalgamated into a health-related benefit, the Work Assessment Allowance (WAA) [17]. WAA is distinguished from its predecessors by a relatively liberal eligibility criteria that gives individuals both with and without prior labour market attachment access to the entirety of NAVs vocational rehabilitation services. Previously, access to these benefits required labour market experience.

WAA is available to individuals with at least 50% reduced work capacity, who possess the potential to regain work function after a period of rehabilitation [17]. Eligibility also requires a medical diagnosis in accordance with either International Classification of Diseases (ICD 10 or 11) [18, 19] or International Classification of Primary Care (ICPC-2) [20]. WAA covers 66 percent of the client’s average income, up to a salary cap of 6 times the National Insurance Scheme basic amount (G).^1^ If an individual has no or insufficient previous income, they receive a minimum, age-dependent sum between Norwegian Kroner (NOK) 242 590—309 621. Clients can combine WWA with up to 22.5 h (60 percent) normal work and the benefit amount is adjusted in accordance with how many hours one works.

As a general rule, one can now receive WAA for up to three years, with the possibility of extension to five years if deemed appropriate by NAV [17]. Prior to January 2018, the maximum benefit period was four years. During this period, recipients must be engaged in an individualised vocational rehabilitation and treatment ("return-to-work") plan for 37.5 h per week. The return-to-work plan is based on medical information from the recipient’s general practitioner (GP), NAV-client interviews and a work capacity assessment. WAA can be revoked if recipients fail to fulfil the conditions outlined in their return-to-work plan. If rehabilitation and treatment measures do not improve the clients work functioning and their work incapacity is deemed permanent, they can then be granted a disability benefit.

Mental and behavioural disorders constitute over 70% percent of all WAA diagnoses for recipients aged 18–29 [21]. NAV divides WAA mental diagnoses into three broad categories:

1. Mild mental conditions (e.g. mental symptoms, stress, sleep problems)—21.6 percent
2. Anxiety or depressive symptoms or conditions—27.0 percent
3. Other mental/behavioural conditions (e.g. psychotic or organic disorders)—22.0 percent

The proportion of young WAA benefits granted due to mental and behavioural disorders has been increasing steadily, foremost due to expansion of the "mild mental conditions" category [21].

Disability benefits follow a similar pattern. Since WAA was introduced, there has been a strong increase in young disability pension incidence, primarily due to rising numbers of 25–29-year-olds being granted disability pensions for behavioural, anxiety and depressive disorders [22]. The majority of this group (85.9%) transition to disability benefits from WAA [23]. The influx into disability benefits among people with severe mental disorders, such as schizophrenia, organic disorders or drug-related disorders, has been relatively stable [22]. In Norway, transitions from disability pension dependency (back) into the labour market are rare and it is therefore considered a permanent state [3].

### Controversy and conflicting claims

WAA was expected to improve labour market outcomes for vulnerable individuals at risk of permanent labour market exclusion. However, the media soon labelled WAA "a waiting room for disability pension" and predicted that there would be a "disability pension bomb" after several years, when the first round of WWA recipients maximised their allotted period of health-related rehabilitation [24]. The OECD backs up these sentiments, claiming that the possibility of transferring from WAA into permanent disability benefits after several years’ compromises rehabilitation efforts [3].

On the contrary, WAA proponents refute the "waiting room" and "disability pension bomb" analogies and provide evidence that WAA has generally improved labour market outcomes for all recipients of health-related rehabilitation benefits, regardless of how long they are in the scheme and irrespective of age [24]. They report that 1) the "new" WAA recipients have a lower probability of transitioning to a disability pension, compared with those who transferred into WAA from older benefits (temporary disability, vocational rehabilitation and medical rehabilitation); and 2) the later an individual entered the WAA scheme, the lower the probability that they would transition to disability pension.

However, WAA may not have improved labour market outcomes in our study population. As mentioned previously, data from NAV shows a sharp spike in disability pension incidence among those aged 25–29 after WAA was introduced, primarily due to behavioural, anxiety and depressive disorders [22].

### Study aims

Preventing early labour market exclusion is top priority both in Norway and internationally [1]. However, there is a knowledge gap regarding the work and welfare trajectories of high-risk young people who have not yet transitioned to permanent disability pension. In order to understand young welfare dependency, we need to further investigate this vulnerable group. Thus, our study addresses three main empirical questions:

1. What were the most typical health-related rehabilitation trajectories for young Norwegians aged 23–27 between 2004–2019?
2. Did the trajectories and composition of health-related benefit recipients change overtime?
3. In parallel with the welfare reform, do we see improved labour market outcomes in our study population?

The basis for our study population is the increasing numbers of 25–29-year olds on permanent health-related disability benefits after WAA was introduced. This group would have started WAA at age 23–27 and then maximised their allotted period of health-related rehabilitation around age 25–29. At this point, if deemed to have permanently decreased work capacity, they would have been eligible for a disability pension.

## Contextual background for the study

### Economic transformations

Since the 1990’s, structural transformations in the Norwegian economy have had negative consequences for adults with low educational attainment. Increased uptake of tertiary education, technological advances and automation have raised the demand for non-routine, high-skilled labour [25] and dramatically reduced the number of jobs that do not require formal academic qualifications [9]. In addition, opening up of the common European labour market has put a pressure on employment opportunities, particularly for Norwegians working in low-wage, unskilled positions [26].

### Young welfare dependency and mental health conditions

While our study does not include diagnostic information, we place an emphasis on mental health problems because, in Norway, most health-related welfare dependency among young people is due to these conditions [27]. There is limited data regarding secular trends in the prevalence of mental disorders in Norway [28]. However, in parallel with increasing welfare dependency due to mental and behavioural disorders there has been a considerable rise in self-reported mental health problems among Norwegian adolescents and young adults [29], as well as a concurrent rise in the proportion of young people receiving treatment for mental health problems from primary care [29].

### Medicalisation

Throughout Europe, policymakers in Europe are increasingly concerned about the medicalisation of unemployment, as it significantly burdens society [30]. This is also the case within the Norwegian context, where the combination of economic transformations, welfare system reforms and healthcare seeking behaviours may have created a paradigm shift towards medicalisation of young people’s labour market struggles. Policy makers, analysts and professionals with field expertise provide different (but complimentary) explanations for this phenomenon [6].

NAV advisors from the Norwegian Social Insurance Medical Association maintain that WWAs medical eligibility criteria may be a culprit. Utilising mild diagnoses, combined with a lack of non-medical-based benefit options, may result in many young people receiving a health-related benefit (WAA) even though the source of their work incapacity is primarily non-medical related problems [31]. Along the same lines, sociologists provide evidence that health-related selection out of the labour market is especially prominent for people with disadvantaged social backgrounds, particularly in Western societies [32]. Economic research provides further evidence for medicalisation of young people’s social challenges [33] and, economic theory suggests that people tend to take on a "disability identity" when the advantages of that role outweigh those gained from employment [34].

### Expectations

Given the large-scale welfare reform and economic transformations, welfare system reforms and changes in health-seeking behaviours that occurred throughout the study period; we expect to see transformations in the background characteristics of our study population across cohorts related to formal academic qualifications. More specifically we predict an increasing proportion of early school leavers over the observation period, as Norway's labour market becomes progressively skills-biased and trends in young people's mental health deteriorate.

We also anticipate that there will be inter-cohort differences related to the NAV reform. In accordance with evidence from previous research, we expect to see progressively fewer transitioning into disability benefits across cohorts [24]. Regarding gender, we expect a greater proportion of women across all cohorts as women generally have a higher uptake of health-related rehabilitation benefits than males [35].

## Methods

### Data source

We used comprehensive longitudinal register data collected and linked by Statistics Norway. These data encompass the entire Norwegian population and contains extensive information on demography, welfare benefits, income and educational activity. The rich and detailed information, stretching over a long time-span makes these data well suited for sequence analysis and depiction of life courses.

### Analytical sample and cohorts

The study population was Norwegian inhabitants aged 23–27 years in either 2004 (cohort 1), 2008 (cohort 2), 2011 (cohort 3) or 2014 (cohort 4), who were granted health-related rehabilitation benefits during a three-month period prior to their cohort start-date. We followed each cohort for six consecutive years. The age restriction provided us with a total population of 3,384 individuals: 638 from cohort 1; 761 (cohort 2); 780 (cohort 3); and 1,205 (cohort 4).

The rationale behind the time periods for our cohorts are based on findings from previous WAA research [12] that: 1) the "new" WAA recipients have a lower probability of transitioning to a disability pension compared to those who transferred into WAA from older benefits (temporary disability, vocational rehabilitation and medical rehabilitation); and 2) the later an individual entered the WAA scheme, the lower the probability that they would transition to disability pension.

Thus, cohort 1 (01.2004—12.2009) represents the pre-NAV reform group, who received temporary disability, vocational rehabilitation or medical rehabilitation benefits. Cohort 2 (01.2008—12.2013) consists of those who transferred from the old benefit scheme into WAA in 2010. Cohort 3 (01.2011—12.2016) are the "new" WAA recipients, who are purported to have a lower probability of transitioning to a disability pension, compared to cohort 2. Finally, based on the claim that, the later an individual entered the WAA scheme, the lower the probability that one would transition to disability pension [12], cohort 4 (01.2014—12.2019) should have the lowest probability of transitioning to disability pension.

### Study design

Typical work and welfare trajectories were identified using multichannel sequence analysis and cluster analysis. Multichannel sequence analysis was performed utilising the approach of Pollock, 2007 [31] adapted and made available in TraMineR by Gabadinho et.al. 2011 [36].

Much of the research on transitions within education, work and welfare state benefits has focused exclusively on single transitions or outcomes [37]. These approaches have some limitations as they focus on single transitions without taking into consideration how these transitions are interconnected and constitute longer parts of the life course. In contrast, here we have utilised a holistic explorative approach, thereby placing our study within a less widespread, but fast growing, field of research – sequence analysis.

This perspective has two main advantages that separates it from conventional methodological approaches within this field of research. Firstly, it adopts a holistic perspective, thus seeing transition between social statuses as interconnected and forming part of a longer life course. Consequently, the dependent variable is not a single state or transition, but a sequence of events. This allows us to shed light on how statuses and transitions are interconnected and combined. Secondly, sequence analysis is an explorative approach that allows the identification of patterns and regularities in data where the analyst has little or no previous knowledge. Regarding the latter, we have extensive knowledge on the use of welfare state benefits, but little knowledge of how they are combined and interconnected.

An important motivation for the multi-channel approach was the question of whether rehabilitation and training allow and facilitate work activity, both in parallel and independent from welfare-state benefits. To enable such analyses, we defined two separate status channels, where each channel consists of mutually exclusive states (Table 1). Thus, instead of observing work and welfare as separate and mutually excluding processes, the current approach allows us to observe how such processes develop and interact over time and cohorts.

The distances between the sequences were calculated using the longest common subsequence (LCS). Our interest here, when studying work and welfare trajectories, lies less with the exact timing of the states than with the actual states and the order of the distinct states experienced. In consequence, our research interest guides us in the direction of a cost-setting scheme that emphasises the number of common attributes between sequences and puts less emphasis on the exact timing of states. In accordance with this, we have chosen to calculate LCS, which emphasises order over timing. Cluster analysis was performed using a two-step approach suggested by Studer 2013 [38],where hierarchical clustering (Ward) was used to provide starting values for partitioning around medoids (PAM) clustering. Clustering quality was assessed using the average Silhouette coefficient repeated over a various number of clusters. The best cluster solution produced six distinct trajectory types. In the last step, we ran multinomial logistic regression on the relationship between sociodemographic variables and the six trajectory types identified by the cluster analysis. For ease of comparison, the regression-based results were presented as average marginal effects (AME). The AME-coefficient provides the effect on the probability of an outcome. Thus, it depicts the average change in probability of a certain outcome when the independent variable increases by one unit.

### Status alphabet

Monthly statuses were specified for a total of six years for each of the four cohorts. At the top of Table 1 under the caption “Channel 1”, we have placed *disability pension.* This is considered a permanent state within the Norwegian benefit system. It presupposes at least 50% reduced work capacity. In the current analysis, *disability pension* takes precedence over all other simultaneous statuses. *Health-related rehabilitation* is a collective term for all public health-related benefits and schemes meant to bring people with impaired health back into work. Over the period of this study, several schemes were introduced, some of which were subsequently replaced. To ensure comparability over the relevant period, these were combined into one broad category. *Social assistance* is the last-resort safety net of the Norwegian welfare state. The benefit is means tested and is intended to be a short-term solution. The *sickness allowance* is a contributory state benefit for individuals who have worked for at least four weeks. To qualify for the sickness benefit, documentation from a medical doctor is required. *Unemployed and occupational handicapped* is a status given to unemployed people waiting for rehabilitation or who had been assessed as having reduced working capacity by NAV. After the introduction of work assessment allowance in 2010, most persons in this group were transferred to WAA and unemployment was no longer considered their main status. The category *Unemployed* has been assigned to those who have registered as ordinary unemployed at their local NAV office. The category includes both those with earned rights to unemployment benefit and those without. *No benefits* is a status assigned to persons with no registered social welfare benefit.

Complementing the statuses in Channel 1 are statuses related to earnings and education. These can be found under the caption “Channel 2” in Table 1. Earnings were drawn from Norwegian tax registers. Four earnings-based quartiles were generated using records for the full workforce aged 16–66 as a starting point for the division into earnings-based groups. In addition to the four earnings-based quartiles, we recorded educational activity. For the status alphabet, educational activity was registered if the current month was in a year with a valid educational record. In cases where both work and positive earnings were recorded at the same time, the current earnings-status is given preference. Months with no registered earnings or education has been labelled “*No work or education*”.

**Table 1:** Status alphabet

Status	Description
**Channel 1**	
Disability pension	Registered with disability pension current month
Health-related rehabilitation benefits	Registered with either temporary disability benefit, vocational or medical rehabilitation, or work assessment allowance current month
Social assistance	Registered with means tested social assistance benefits current month
Sickness allowance	Registered with sickness allowance current month
Unemployed, occupational handicapped	Registered as unemployed and occupational handicapped / reduced working capacity current month
Unemployed, ordinary	Registered as ordinary unemployed current month
No benefits	No benefits registered current month
**Channel 2**	
Income Q1	Monthly status is based on annual earnings in the 1^st^ quartile (age 16–66)
Income Q2	Monthly status is based on annual earnings in the 2nd quartile (age 16–66)
Income Q3	Monthly status is based on annual earnings in the 3rd quartile (age 16–66)
Income Q4	Monthly status is based on annual earnings in the 4th quartile (age 16–66)
Education	Registered under education current month if month is in a year with a valid educational record and none of the above monthly statuses apply
Not in work or education	Registered if none of the other statuses in Channel 2 apply current month

## Results

### Descriptives

In Table 2, we present and compare status durations in months. In channel one, we find that people in the first cohort spent around half the amount of time on health-related rehabilitation benefits compared to later cohorts (Table 2). In addition, the average amount of time spent on disability pension increased by almost 6 months between the first and last cohorts (Table 2). Increased time on health-related rehabilitation benefits and disability pension was accompanied by a simultaneous decrease in the average number of months spent occupational handicapped unemployed or without any registered welfare benefits (Table 2).

Descriptive results for channel two are more stable across cohorts compared to channel one (which runs on a parallel time-line). Participation in education and normal employment (Q1 level income) increased by 0.4 months and 3.8 months respectively. The average number of months spent as normal unemployed also rose by 1.3%, while normal employment (income levels Q2-Q4) and the "no work or education" status decreased slightly overtime (Table 2).

**Table 2:** Cohort-specific status duration in months

Cohort-specific status duration in months:	2004-cohort	2008-cohort	2011-cohort	2014-cohort
**Channel 1**				
Disability pension	3.1	3.7	4.6	8.8
Health-related benefits	17.7	32.8	41.1	37.5
Social assistance	6.8	4.5	3.2	3.4
Sickness allowance	3.6	3.2	2.6	2.0
Unemployed, occupational handicapped	24.7	12.0	8.2	7.8
Unemployed, ordinary	11.2	15.7	12.3	12.5
No benefits	5.1	0.5	0	0
Total (N)	72 (638)	72 (761)	72 (780)	72 (1,205)
**Channel 2**				
Income Q1	21.3	21.3	24.0	25.1
Income Q2	11.3	11.3	11.2	10.3
Income Q3	3.9	3.8	3.6	2.6
Income Q4	1.2	1.6	1.3	0.7
Education	4.0	4.1	4.9	4.4
No work or education	30.4	29.9	27.0	28.9
Total (N)	72 (638)	72 (761)	72 (780)	72 (1,205)

In Table 3, we look at distribution and changes in distribution of socioeconomic characteristics. Over the observation period, the proportion of early school leavers increased 6.9% from 55.0% to 61.9% (Table 3). The study population also became steadily more ethnically diverse across cohorts, mainly due to a 4.5% increase in individuals from Non-western countries (Table 3). Women are overrepresented in all cohorts, however the proportional difference between genders remained fairly stable over time (Table 3). Further, turbulence (Table 3) i.e. the number of distinctive sequences and the time in each state, dropped from 8.8 to 6.7 between the first and last cohort.

Between the first and last cohorts, the share of WAA recipients with parents who were not dependent on disability benefits increased 8.9% from 48.0% to 56.9%, accompanied by an equivalent percentage decrease in those with one disabled parent (Table 3). The proportion with two disabled parents remained stable overtime. On average parents had completed compulsory primary and lower secondary education as well as some basic upper secondary education. Over the observation period the duration of parental upper secondary education increased from 3.2 (7 to 12 months) to 3.5 (> 25 months).

**Table 3:** Descriptives

Cohort-specific descriptive statistics (%)	2004-cohort	2008-cohort	2011-cohort	2014 cohort
Turbulence (mean) ^a^	8.8	7.9	6.4	6.7
Country background				
Norway	80.9	80.2	78.9	74.3
Western Europe, North-America,	10.2	8.7	8.3	12.3
Oceania				
Non-western	8.9	11.2	12.8	13.4
Gender				
Male	45.9	46.4	51.5	47.6
Female	54.1	53.6	48.5	53.3
Education				
Finished upper secondary education	45.0	39.8	39.2	38.1
Early school leaver	55.0	60.1	60.2	61.9
Region				
Urban	78.7	81.4	80.1	82.8
Rural	21.3	18.6	20.0	17.2
Parental disability pension status				
No parental disability pension	48.0	48.0	50.0	56.9
One disabled parent^b^	38.4	39.2	38.1	29.5
Two disabled parents	13.6	12.9	12.1	13.6
Parental education NUS level (mean)c	3.2	3.2	3.3	3.5

a Turbulence is a composite measure reflecting the number of distinctive sequences and the time in each state [34]. Higher turbulence implied shorter spells and more shifts between statuses

b For the purposes of the operational definition of "disabled parent" is having a parent who is dependent on health-related disability benefits

c Parental education was measured for the parent with the longest education in years using the Norwegian Standard Classification of Education (NUS2000) [39] normalised from 0 to 1 for the presentation of average marginal effects

### Trajectories

Next, we present the most typical trajectory types resulting from LCS matching and clustering procedures along with the trajectory-specific risk factors depicted with average marginal effects (AME) (Appendix Fig. 7 and Table 1). Table 4 provides an overview of trajectory types and their defining characteristics. Our analyses identified six distinct clusters.

**Table 4:** Trajectory types and their risk factors

Type	Trajectory	Trajectory risk factors
Health-related rehabilitation + No work or education—C1 (n = 783)	Channel 1: long period of health-related rehabilitation	Early school leavers ↑ 4.0% (p < 0.005) Parental education below average ↑ 10.4% (p < 0.05) 2008-cohort membership ↑ 24.8% (p < 0.001) 2011-cohort membership ↑ 17.1% (p < 0.001) 2014-cohort membership ↑ 10.4% (p < 0.001) i
	Channel 2: no work or education activity	
Unemployed O.H, low income work (Q1, Q2) + sickness absence—C 2 (n = 416)	Channel 1: short spell of health-related rehabilitation → occupational-handicapped unemployment + sick leave	2008-cohort membership ↓ 9.6% p(< 0.001) 2011-cohort membership ↓ 9.4% p(< 0.001) 2014-cohort membership↓ 10.4% p(< 0.001)
	Channel 2: Q1-level income employment → Q2 income-level work	
Normal unemployment, unstable income + sickness absence—C 3 (n = 648)	Channel 1: short-spell of health-related rehabilitation → sickness absence	Female ↓ 3.0% (p < 0.05) Early school leaver ↓ 9.2% (p < 0.001) Two disabled parents ↓ 9.4% (p < 0.001)
	Channel 2: normal unemployment + low income work (Q1, Q2)	
Health-related rehabilitation + low income work (Q1)—C4 (n = 673)	Channel 1: long period of health-related rehabilitation	Female ↑ 9.0% (p < 0.001) Parent education above average ↑ 8.6% (p < 0.05) 2008-cohort membership ↑ 8.4% (p < 0.001) 2011-cohort membership ↑ 14.6% (p < 0.001) 2014-cohort membership ↑ 14.8% (p < 0.001)
	Channel 2: low income (Q1) work	
Unemployment O.H, social assistance + No work or education—C 5 (n = 280)	Channel 1: short health-related rehabilitation → long occupational unemployment	Male ↑ 2.3% (p < 0.05) Early school leaver ↑ 2.3% (p < 0.05) Urbanicity ↑ 2.7% (p < 0.005) 2008-cohort membership ↓ 2.3% (p < 0.001) 2011-cohort membership ↓ 2.6% (p < 0.001) 2014-cohort membership ↓ 2.6% (p < 0.001)
	Channel 2: no work or education	
Disability pension + No work or education—C 6 (n = 584)	Channel 1: long health-related rehabilitation → disability pension	Early school leaver ↑ 4.0% (p < 0.005) Two disabled parents ↑ 9.2% (p < 0.001) 2014-cohort membership ↑10.3% p (< 0.001)
	Channel 2: no work or education	

### Chronograms and sequence index plots

In Appendix Figs. 1, 2, 3, 4, 5 and 6, we present summary graphs called chronograms (right) and individual sequences (left) of the two channels under study for each of the six trajectory types. Importantly, both channels unfold simultaneously over the same time axis, allowing us to scrutinise how work and welfare trajectories interact and develop over time. In the sequence index plots to the left, individual sequences are horizontal stacked bars across the x-axis [40]. The x-axis represents time, and each stacked bar along the y-axis represents one person-sequence. Colours indicate different states depicted by the labels between each plot pair.

We have sorted the sequence index plots using multidimensional scaling (MDS) to facilitate interpretation. To the right, the state distribution plots (chronograms) represent the aggregate distribution of states at any month of the observation time and provide summary information for the entire sample of sequences [40]. Notably, the latter does not allow the extraction of information about individual sequences as the sequence index plots do.

### Trajectory types and risk factors

#### Health-related rehabilitation + No work or education—C1 (*n* = 783, 23.1%)

Here we find a trajectory dominated by a long period of health-related rehabilitation without any simultaneous work or education activity. The proportion following this trajectory jumped 25.4% between the first and second cohorts from 8.2% to 33.6%, then decreased slightly in the later cohorts, 27.1% (2011) and 31.2% (2014). This phenomenon is reflected in the corresponding chronogram (Appendix Fig. 1) where we see a sharp spike in health-related rehabilitation after approximately 2.5 years, coinciding with the introduction of WAA in March 2010.

Regression analyses (Appendix Fig. 7) found that early school leavers had a 4.0% (*p* < 0.005) increased chance of following the trajectory compared with those who completed upper secondary school. In addition, the trajectory-probability decreased 10.4% (*p* < 0.05) if one's parents had a relatively low level of education. Compared to the 2004-cohort, the 2008-cohort had a 24.8% (*p* < 0.001) increased chance of following this trajectory, while 2011-cohort membership inferred 17.1% (*p* < 0.001) increased risk and the 2014-cohort had 10.4% (*p* < 0.001) increased risk.

### Unemployed O.H, low income work (Q1, Q2) + sickness absence—C 2 (*n* = 416)

This cluster includes predominantly subjects with sequences that included a short spell of health-related rehabilitation before transitioning into occupational-handicapped unemployment, interspersed with periods of sick leave (Appendix Fig. 2). This trajectory also included a fair amount of work activity. Initially Q1-level income employment dominated but over time was surpassed by Q2 income-level work, and even some Q3-Q4 level employment. AMEs depicted in Appendix Fig. 7 that individuals in the 2004-cohort had around a 10.0% higher trajectory chance of following this trajectory than those in the later cohorts.

### Normal unemployment, unstable income + sickness absence—C 3 (*n* = 648)

Here we have a trajectory consisting of general instability (Appendix Fig. 3). After short-spell of health-related rehabilitation, individuals in this cluster mainly transitioned into alternating periods of normal unemployment, low income work (Q1, Q2) and sickness absence.

The proportion following this trajectory increased steadily over the observation period, from 17.3% in the first cohort to 33.6% in the last cohort. Being female or an early school leaver (reference upper secondary school completion) decreased the trajectory probability by 3.0% (*p* < 0.05) and 9.2% (*p *< 0.001) respectively (Appendix Fig. 7). Having two disabled parents decreased the risk of following this trajectory by 9.4% (*p* < 0.001) compared to those with parents who were not dependent on disability benefits.

### Health-related rehabilitation + low income work (Q1)—C4 (*n* = 673)

This trajectory, made up predominantly of subjects who participated in a long period of health-related rehabilitation punctuated by intervals of normal unemployment, occupational handicapped unemployment and/or low income (Q1) work (Appendix Fig. 4). Similar to the ¨Health-related rehabilitation + No work or education trajectory we see a sharp spike in health-related rehabilitation participation (Appendix Fig. 4), corresponding to the initiation of WAA in 2010.

Over the observation period there was a fivefold increase in the proportion following this trajectory, 8.9% in the 2004-cohort vs 43.8% in the 2014-cohort. AMEs (Appendix Fig. 7) also show that the risk of following this trajectory increased overtime. Compared to the 2004-cohort, belonging to the 2008-cohort inferred 8.4% (*p* < 0.001) increased risk, followed by 14.6% (*p* < 0.001) and 14.8% (*p* < 0.001) in the 2011- and 2014-cohorts respectively. In addition, being female increased the trajectory probability by 9.0% (*p* < 0.001), while having parents with a relatively high level of education inferred 8.6% increased risk (*p* < 0.05).

### Unemployment O.H, social assistance + No work or education—C 5 (*n* = 280)

Individuals in cluster 5 generally had sequences involving a short stint of health-related rehabilitation, followed by long periods of occupational unemployment and no work or education (Appendix Fig. 5).

The proportion following this trajectory decreased almost fivefold over the observation period: 64.3% (2004), 13.2% (2008), 8.9% (2011) and 13.6% (2014). Urbanicity (reference rural dweller) and being male (reference female), each increased the trajectory probability by 2.7% (*p* < 0.005). In addition, being an early school leaver inferred 2.3% (*p* < 0.05) increased risk compared to those who completed upper secondary school (Appendix Fig. 7). Compared to the 2004-chort, belonging to the 2008- cohort was associated with a 2.3% (*p* < 0.001) decreased chance of following this trajectory, while cohort 2011 or 2014 membership each entailed 2.6% decreased risk (*p* < 0.001) (Appendix Fig. 7).

### Disability pension + No work or education—C 6 (*n* = 584)

Here we find those who transitioned, for the most part, onto disability pension dependency and no work or education after a long spell of health-related rehabilitation (Appendix Fig. 6). The proportion following this trajectory increased more than threefold over the observation period, 15.9% in the 2004-cohort; 14.6% (2008-cohort); 19.5% (2011-cohort) and 50.0% (2014 cohort-cohort). In addition, having two disabled parents (reference no disabled parents) increased the trajectory probability 9.2% (*p* < 0.001) while high-school dropout (reference upper secondary school completion) inferred 4.0% (*p* < 0.005) increased risk (Appendix Fig. 7).

## Discussion

Our study addressed three main empirical questions:

1. What were the most typical health-related rehabilitation trajectories for young Norwegian inhabitants aged 23–27 between 2004–2019?
2. Did trajectories and composition of health-related benefit recipients change over the observation period?
3. In parallel with the welfare reform, do we see improved labour market outcomes in our study population?

Preventing early labour market exclusion is top priority in Norway and internationally, however little is known about the health-related rehabilitation trajectories of high-risk young people who have not yet transitioned into permanent disability pension. An important priority for our research is to help close this knowledge gap.

Our study population is, in general, a vulnerable group with poor labour market outcomes. The majority are early school leavers, following disadvantaged trajectories consisting of welfare dependency, unemployment, minimal educational activity and unstable, low-income work. We identified six broad trajectory types, and while some could be considered less "problematic" than others, almost no-one in the study population ended up self-sufficient through work.

### Reclassification of welfare statuses and medicalisation

Over the study period we witnessed considerable increase in the proportion following trajectories dominated by long periods of health-related rehabilitation (Clusters 1, 4), accompanied by a substantial decline in the share following the unemployment O.H trajectory (Cluster 5). Furthermore, the average time spent in health-related rehabilitation increased by nearly twenty months while the average duration of "occupational handicapped unemployment" decreased by nearly seventeen months.

What we could be observing here is an administrative re-categorisation of unemployed people waiting for rehabilitation. After WAA was introduced, many individuals categorised as "Occupational handicapped unemployed" were instead provided with a primarily health-related status. It is possible that the increased disability pension incidence over the observation period can partly be attributed to this reclassification of welfare statuses. Giving people a dominant label of poor health, rather than a status related to absence of work, may have the unfortunate effect of steering people into permanent disability benefits (which are also primarily focused on poor health).

#### Societal perspective

Policymakers in Europe have expressed growing concerns about the medicalisation of unemployment, as it significantly burdens society [30]. In Norway, the combination of economic transformations, welfare system reforms and healthcare seeking behaviours may have created a paradigm shift towards medicalisation of young people’s labour market struggles [6]. Particularly troubling is the high incidence of young individuals receiving disability pensions, with historical data indicating low success rates for reintegration into the workforce [3]. This situation not only imposes substantial financial obligations on the government regarding social security payments but also deprives society of the valuable contributions these young individuals could have made.

Recent research in this field indicates that the medicalisation of unemployment has become more prevalent [41–43]. This trend can be partly attributed to better understanding the unemployed people’s health challenges. However, there is evidence that strict eligibility requirements for non-medical benefits [44] increases the emphasis on illness or disability as justifications for accessing benefits or being exempted from certain obligations [45–47] This could be a contributing factor in Norway where unemployment benefits are only available to those who have earned the right through work. The only non-medical benefit option available to individuals without previous work experience is a meagre, means-tested social assistance benefit, considered the last safety net in the Norwegian welfare system.

As welfare reforms worldwide move towards consolidated, one-stop-shop services and eligibility criteria for non-medical benefits become stricter, there is a concerning possibility that a growing number of marginalised young adults could be reclassified as ill and receive health-related benefits. Such a trend could increase permanent welfare dependency and labour market exclusion among this group internationally.

#### Individual perspective

While increasing health-related welfare dependency impacts society negatively, individuals reliant on health-related welfare may perceive it more favourably. This perception could be due, in part, to the reduced stigma associated with being categorised as sick rather than unemployed. Social legitimacy research on welfare benefits has shown that society perceives sick persons as more deserving than unemployed individuals [48]. Moreover, the medicalisation literature suggests that sickness relieves individuals from social role obligations, which helps justify inactivity and benefit receipt [30].

Furthermore, our study found that individual sequences became less turbulent over time, indicating increased stability. This stability resulted from less shifting between low-income employment and unemployment statuses accompanied simultaneously by longer spells spent in health-related rehabilitation, and increased uptake of disability benefits, which often represent a stable and generally permanent state. While previous research consistently highlights the health benefits of being employed compared to being jobless, evidence also suggests that poor working conditions can deteriorate one's health. Current trends towards work fragmentation and flexible labour markets [49] have negatively impacted low-skilled young people [50], trapping them in low-paid, insecure work and unemployment cycles [51]. Job insecurity poses a comparable threat to health as unemployment [52], emphasising that societal perspectives of what is beneficial may not always align with the best-case scenario for individuals.

### Work participation

Between the first and last cohort we observed a 16.3% increase in the proportion of individuals following the least problematic trajectory, characterised by normal unemployment, unstable income, and sickness absence (Cluster 3). However, regression analyses found no significant association between the trajectory probability and a specific cohort. Over the observation period, the average time spent with any income remained relatively stable, increasing by only one month, while the average time spent in "no work or education" decreased by one-and-a-half months.

At first glance, this is a rather unintuitive finding. Given the large-scale economic transformations and increased proportion of early school leavers that occurred over the observation period, we expected to see decreased work activity overtime. This suggests that WAA caters to different individuals than the previous health-related rehabilitation benefits, which makes sense given that composition of health-related benefit recipients changed across cohorts.

### Compositional changes

#### Parental factors

We discovered a decline in parental disability pension dependency over time, while the average level of parental education improved. These findings could indicate that individuals on WAA came from increasingly less disadvantaged social backgrounds, which may have brought them closer to the labour market than their predecessors.

The decreasing prominence of intergenerational welfare transmission is an intriguing finding, running counter to current international and Norwegian literature on the topic [53, 54] and requires further exploration. However, it is also important to note that the trajectory ending in permanent disability pension (Cluster 6) was significantly associated with having two parents who were disability pensioners. Moreover, the proportion of individuals following this trajectory tripled over the observation period. Interestingly, the trajectory probability was not associated with parents having a below average level of education.

#### Educational attainment, country background and mental health problems

We also find evidence that, in some aspects, young people on health-related rehabilitation benefits are becoming more disadvantaged overtime. The role of education in determining employment and income prospects for young individuals is crucial [55], for example incomplete upper secondary education increases the risk of long-term exclusion from the labour market [55]. Our study reveals a concerning trend of a higher proportion of early school leavers over time, indicating a growing educational disadvantage across cohorts. Additionally, we found a significant association between high school dropout and following a trajectory leading to a disability pension dependency.

Our study population became more ethnically diverse over time, likely due to the inflow of non-Western immigrants into Norway [56]. This has resulted in an increasing disadvantage based on country background. Individuals from non-European countries tend to have lower educational attainment than native-born individuals and face a higher likelihood of unemployment, especially during challenging labour market conditions [10]. Moreover, they encounter more difficulties in finding new employment opportunities [10].

Existing literature also highlights the significant contribution of mental health conditions to welfare dependency among young adults. Over time, there has been a noticeable rise in self-reported mental health problems among Norwegian adolescents and young adults, accompanied by an increased proportion seeking treatment for mental health issues from primary care services. These findings align with our observations regarding educational status and country background. Young people with mental health conditions are more susceptible to dropping out of education and face substantial obstacles in accessing the labour market [57]. Markussen & Seland, 2012 [58] find that approximately half of early school leavers attribute their dropout to poor mental health.

Individuals with a non-European country background face a double disadvantage concerning education and mental health. Not only do they have a higher risk of leaving school early, but a larger proportion report mental health problems [59]. Furthermore, refugees are more likely to consult their general practitioners about mental disorders than the general population [60]. Several factors contribute to this inequality, including lower socioeconomic status, discrimination based on immigrant backgrounds, language barriers, and exposure to adverse life events [61, 62]. Interestingly however, country background was not a significant risk factor for any trajectory type. Most notably, it did not increase the probability of following the trajectory ending in disability pension.

### Gender

Our analysis also provides evidence that gender plays a role in young people’s work and welfare trajectories. Men are more likely to follow trajectories characterised by unemployment and some labour market activity while women were more likely to participate in long spells of health-related rehabilitation. Women are overrepresented in all cohorts, although the gender gap remained relatively stable overtime.

### Future research

Investigating young health-related rehabilitation trajectories using sequence analysis has provided valuable new insights. The influence of administrative status re-categorisations and our observations regarding the diversification of health-related welfare dependency are intriguing findings that warrant follow-up with causal research.

### Strengths

Using high-quality, register data sets allowed us to bypass the quintessential challenges associated with longitudinal surveys such as low return- and high attrition rates. Furthermore, our data encompasses the entire Norwegian population rather than a representative sample, which makes it possible to study small but important and hard-to-reach groups.

### Limitations

We have identified several limitations. Firstly, our analyses do not include diagnostic information, it would be both interesting and relevant to know if the probability of following a particular trajectory was influenced by one's diagnosis. Another limitation concerns generalisability, the study context is primarily relevant for countries with comprehensive, generous welfare systems and skills-biased labour markets. In addition, due to the descriptive nature of our study, we cannot determine whether observed associations reflect cause-and-effect relationships.

## Conclusion

Norwegian health-related rehabilitation benefit recipients aged 23–27 are a vulnerable, disadvantaged group with suboptimal mental health, functioning and labour market outcomes. Our study reveals that (for this demographic) the NAV reform has not succeeded in its objective of getting "more people into work, fewer on welfare benefits".

Given that welfare reform has not led to the intended life course trajectory changes, our research shows that it is difficult for the authorities to divert life courses in a specific direction. One can therefore question the usefulness of such initiatives, which have exposed individuals to more extended periods of temporary health-related rehabilitation (without any visible improvement in health, functionality or employment outcomes) before eventually funnelling a greater proportion into permanent disability benefits. Comparatively, it would be intriguing to explore whether other European countries have succeeded more in redirecting their citizens' welfare-state trajectories through similar one-stop shop reforms.

## Supplementary Information

**Additional file 1.**

## Figures and Tables

**Fig. 1:**
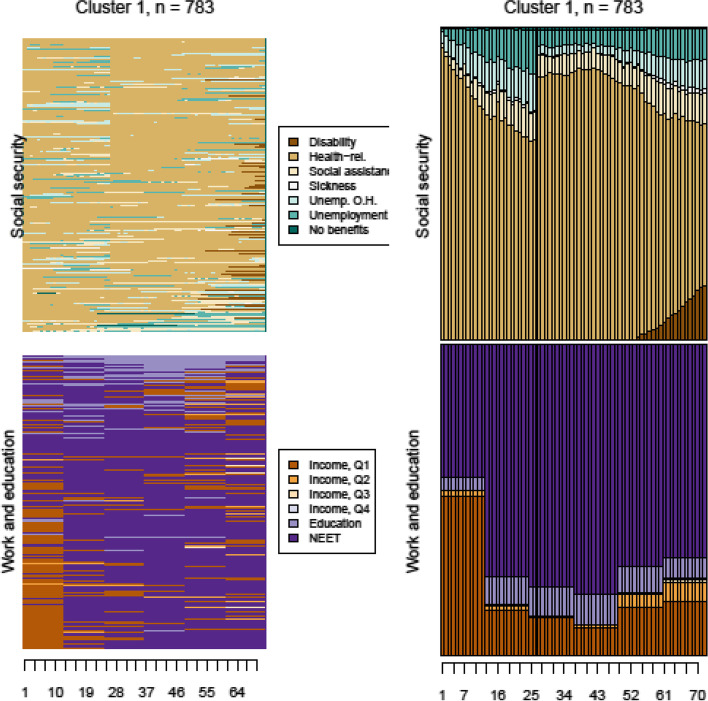
Cluster 1: Sequence plots and chronograms

**Fig. 2:**
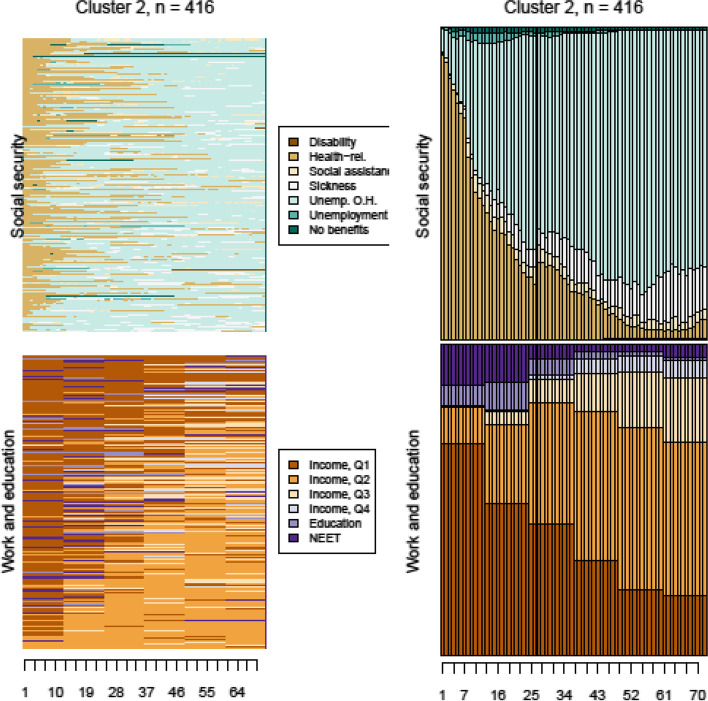
Cluster 2: Sequence plots and chronograms

**Fig. 3:**
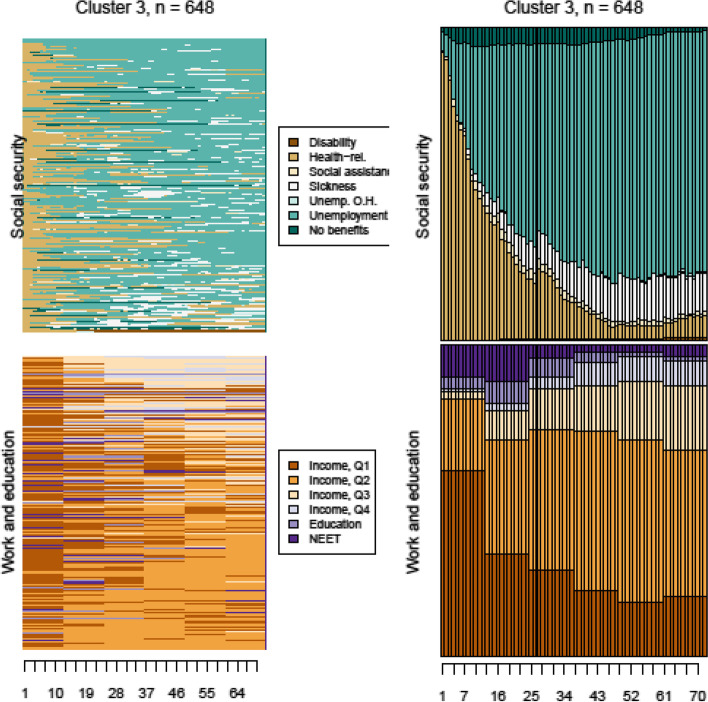
Cluster 3: Sequence plots and chronograms

**Fig. 4:**
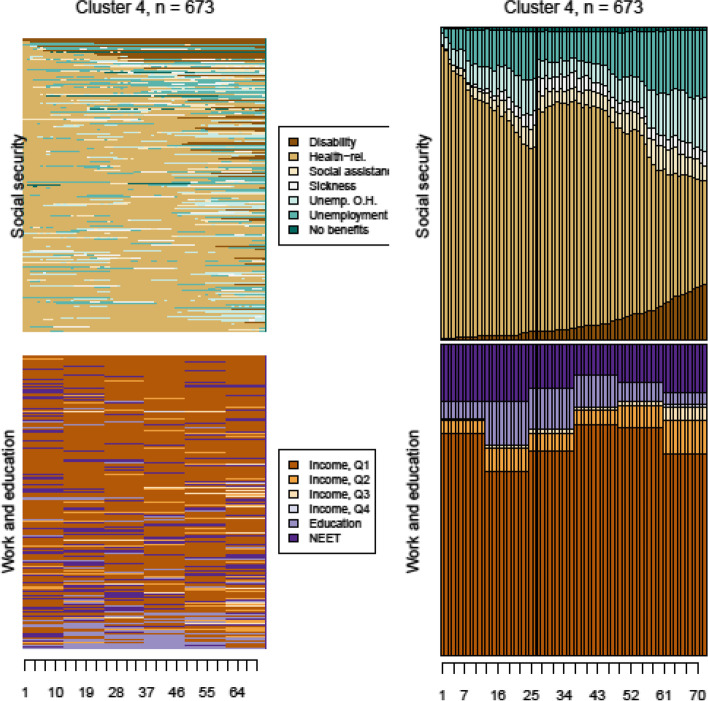
Cluster 4: Sequence plots and chronograms

**Fig. 5:**
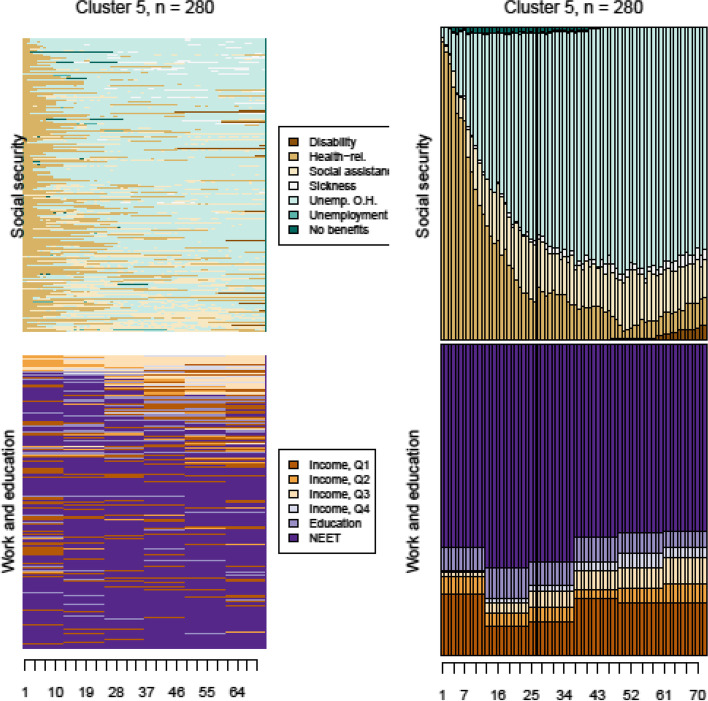
Cluster 5: Sequence plots and chronograms

**Fig. 6:**
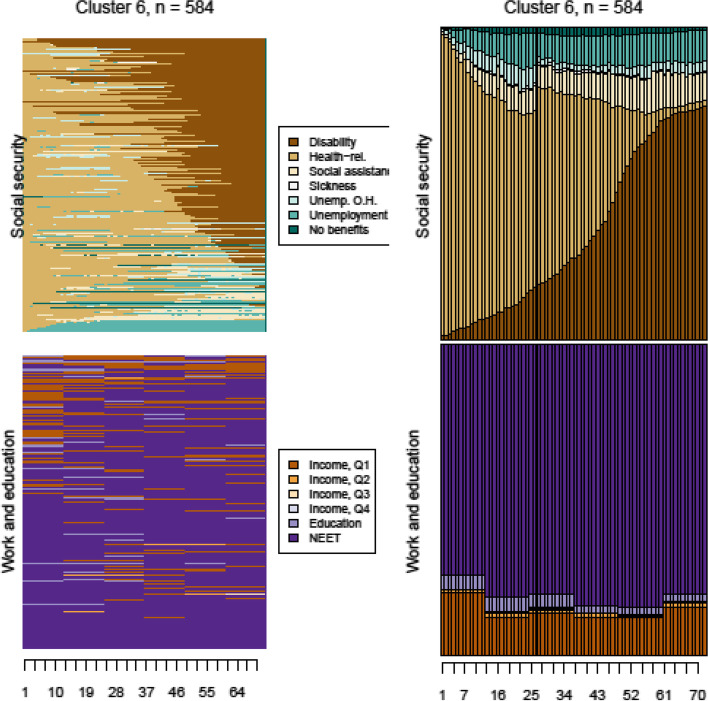
Cluster 6: Sequence plots and chronograms

**Fig. 7:**
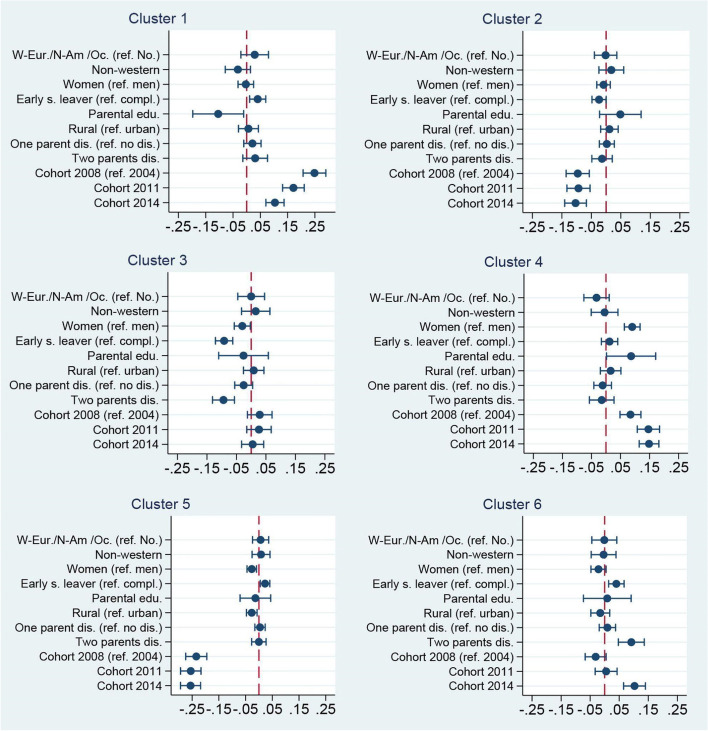
Average Marginal Effects

## Data Availability

The datasets analysed in this study cannot be shared publicly because of Norwegian data protection regulations. Nevertheless, the owners of the data, Statistics Norway, can provide access to the register data. Interested researchers can submit applications to obtain access to the relevant information contained within the public administrative registries in Norway. https://www.ssb.no/en/data-til-forskning/utlan-av-data-til-forskere/soknad

## Abbreviations

WAA: Work Assessment Allowance
NAV: Norwegian Labour and Welfare Administration
OECD: Organisation for Economic Co-operation and Development
ICPC: International Classification of Primary Care
ICD: International Classification of DiseasesIA
NOK: Norwegian Kroner
LCS: Longest common subsequence
AME: Average marginal effects
GP: General practitioner

## Footnotes

1 National Insurance scheme basic amount (G): A standard amount that is used to calculate benefits and pensions, and which is set on 1 May each year. As of 1 May, 2022, the basic amount is NOK 111,477 per year and NOK 9,290 per month.

## Declarations

### Administrative register-based surveys

Statistics Norway collects data from public bodies, such as the Directorate of Taxes and NAV. Large data sets are transferred to Statistics Norway, and according to the Statistics Act, individuals do not have the opportunity to opt-out (i.e. individual consent is not required).

### Survey questionnaires

In addition Statistics Norway has the authority to impose a requirement on individuals to take part in surveys (i.e. individual consent to participate is not required). Written information is always provided to respondents prior to any such survey, with details of what the survey is about, what other data will be linked to the data collected, what the data will be used for, who will have access to the data and when it will be deleted. Details of who has taken part in the survey remain confidential.

According to Sect. 15. of the Statistics Act, Public use of anonymous data—Statistics Norway can give researchers access to data for use in research, and public bodies access to data for statistics and analyses, but only in a de-identified or anonymous form. Statistics Norway is a member of and follows the guidelines of the European Statistical System (ESS). Regulation (EC) No. 223/2009 of the European Parliament and of the Council on European statistics is also known as the European “Statistics Act”. The regulation is also incorporated into the Norwegian Statistics Act. The overarching principles for the production of European statistics are detailed in guidelines (the European Statistics Code of Practice).

Our study does not include data from Norwegian Health registries and therefore we did not require The Norwegian Regional Committee for Medical and Health Research Ethics (REC) to grant exemption from individual consent.

We confirm that all methods were performed in accordance with the guidelines and regulation of the Norwegian National Research Ethics Committees, the Norwegian Centre for Research Data and the Declaration of Helsinki.
