# Supplementary material for: Changes in health-related rehabilitation trajectories following a major Norwegian welfare reform
Source: BMC Public Health. 2023 Jul 28;23:1444. doi: 10.1186/s12889-023-16272-9 (PMC10375644; doi:10.1186/s12889-023-16272-9)
Supplement: Supplementary file 1 — Additional file 1. [file 12889_2023_16272_MOESM1_ESM.docx]

**Appendix Table 1: Average Marginal Effects**

| **Variable** | **Cluster 1** | **Cluster 2** | **Cluster 3** | **Cluster 4** | **Cluster 5** | **Cluster 6** |
| --- | --- | --- | --- | --- | --- | --- |
| World region (ref. Norway) |  |  |  |  |  |  |
| W-Eur./N-AM / Oc. | 0.029431 | -0.0016126 | -0.0000703 | -0.0325932 | 0.0061658 | -0.0013208 |
| Non-western | -0.0322703 | 0.0177355 | 0.0153478 | -0.0049066 | 0.0078695 | -0.0037758 |
| Women (ref. men) | -.0031246 | -.0085453 | -.0302173* | .090146*** | -.026904** | -.0213547 |
| Early s. Leaver (ref. compl.) | 0.0403162** | -0.0237634 | -0.0918223*** | 0.0123066 | 0.0228254* | 0.0401376** |
| Parental edu. | -0.1042811* | 0.0483417 | -0.02604 | 0.0863985* | -0.013263 | 0.0088438 |
| Rural (ref. urban) | .0064161 | .0112782 | .0084994 | .0159744 | -.0269735** | -.0151947 |
| Parental disability (ref. no dis.) |  |  |  |  |  |  |
| One parent disabled | 0.0211052 | 0.0025538 | -0.0254558 | -0.0115474 | 0.0040053 | 0.0093388 |
| Two parents disabled | 0.0310506 | -0.0137005 | -0.093992*** | -0.0146882 | -0.0003001 | 0.0916302*** |
| Cohort (ref. 2004) |  |  |  |  |  |  |
| 2008 cohort | 0.248484*** | -0.096148*** | 0.0289487 | 0.0842334*** | -0.234404*** | -0.0311141 |
| 2011 cohort | 0.1712831*** | -0.0935811*** | 0.0263816 | 0.1459651*** | -0.2549534*** | 0.0049046 |
| 2014 cohort | 0.1036072*** | -0.1035279*** | 0.004934 | 0.1480987*** | -0.2557301*** | 0.1026181*** |

*p (<0.05) **p (<0.005) ***p (<0.001)
